# The safety of a therapeutic product composed of a combination of stem cell released molecules from adipose mesenchymal stem cells and fibroblasts

**DOI:** 10.2144/fsoa-2020-0027

**Published:** 2020-05-29

**Authors:** Greg Maguire, Peter Friedman

**Affiliations:** 1 Bio Regenerative Sciences, Inc. San Diego, CA 92121, USA; 2 Animal BioSciences, LLC Bartow, FL 33830, USA

**Keywords:** inflammation, stem cells, stem cell safety, stem cell secretome, toxicity, tumorigenesis

## Abstract

**Aim:**

We sought to determine the safety profile of a therapeutic candidate composed of the released molecules from a combination of human adipose-derived mesenchymal stem cells and fibroblasts. Although stem cells, their progenitor cells and the molecules that are released from these cells have some demonstrated therapeutic value, much more needs to learn about the efficacy, mechanism of action and the safety profiles of these stem cell-based therapeutics.

**Methods:**

A number of cellular, *in vitro*, *in vivo* and human studies were performed to analyze cellular, tissue and systemic safety profiles of the combinatorial product.

**Results:**

At the levels tested in this study, ranging from demonstrated therapeutic doses to supratherapeutic doses, the combinatorial product demonstrated an excellent safety profile in all *in vitro*, cellular, tissue and systemic studies.

**Conclusions:**

We found evidence that a therapeutic candidate composed of the molecules released from human adipose-derived mesenchymal stem cells and human fibroblasts has an excellent safety profile, and that the product warrants further studies for safety and efficacy where dosing may include topical application, injection and oral application.

## Lay abstract

Stem cell transplants have demonstrated life-saving capabilities for some diseases, and the molecules released from stem cells are currently in therapeutic development for a number of conditions. Stem cell science is a relatively new science and is in need of a better understanding of mechanisms of action and acute and long-term safety profiles. Here, we performed a number of safety tests for stem cell released molecules from a combination of adipose-derived mesenchymal stem cells and fibroblasts that have demonstrated efficacy in a number of conditions. Using *in vitro*, *in vivo* and skin sensitivity studies in humans, the stem cell therapeutic was found to have an excellent safety profile when tested for toxicity, mutagenicity, tumorigenesis, ocular toxicity, inflammation and irritation.

Stem cell transplants have been used for over 40 years with demonstrated life-saving capabilities for some blood diseases [1], and the molecules and exosomes released from the stem cells are currently in therapeutic development for a number of diseases and conditions, including neurodegenerative diseases [2], heart conditions [3], glaucoma [4], hearing loss [5] and skin diseases [6,7]. However, stem cell science is a relatively new science, and therapeutic development using stem cells, even approved stem cell therapies for blood diseases, is in need of a better understanding of mechanisms of action and acute and long-term safety profiles, both for the cells and their released molecules. Approved bone marrow stem cell (BMSC) transplants have many associated risks, including possible induction of cancer [8], including skin cancer [9], the transference of cancer cells from the donor to the patient [10] – given the bone marrow is a site of recirculated cancer cells [11,12] – transference and engraftment of BMSCs with pathogenic mutations [13], and aging of the tissue in which the implant occurs [14]. Many factors, which are often overlooked, must be considered when developing stem cell-based therapeutics, including something as fundamental as the choice of stem cell type where adipose mesenchymal stem cells (ADSCs) have many advantages over BMSCs for therapeutic development [15]. For example, even the molecules from BMSCs may induce cancer [16], whereas from ADSCs do not. Moreover, ADSCs can be used for expressing and delivering targeted therapeutics [17], including the treatment of cancer [18], for example, lung cancer and melanoma [19], osteoarthritis [20] and cardiovascular disease [21].

Here, we performed a number of safety tests for a stem cell-based therapeutic comprised of the stem cell released molecules (secretome) from a combination of ADSCs and fibroblasts (FBs), the combination of which has demonstrated efficacy in a number of conditions [2,7] and is conceptually based on developing a system therapeutic [22] for the physiological renormalization of tissue in various disease states or abnormal conditions [23]. Using *in vitro, in vivo* and skin sensitivity studies in humans, the stem cell therapeutic comprised of stem cell released molecules from ADSCs and FBs was determined to have an excellent safety profile when tested for *in vitro* and *in vivo* toxicity, the Ames mutagenicity assay, *in vivo* tumorigenesis, *in vivo* inflammation, ocular histology and a human skin patch test for irritation and allergic reaction.

## Method

Because a combination of the secretome from ADSCs and FBs has been shown to be efficacious for several conditions [2,7], we performed a number of safety studies for the combination to help clarify its use in humans under acute and chronic conditions.

Stem cell culture procedures have been described previously [2] and performed using the basic procedures described in the patent numbers 9545370 and 9446075. We used a 50–50% mix of the molecules released from the two cell types. All processes were performed using GMP procedures. Briefly, a proprietary collection of stem cell lines derived from human skin were cultured using no penicillin/streptomycin, and 1% fetal bovine serum, under hypoxic conditions. When cultures reached confluence, they were passaged for a limited number of times (fewer than 10) before disuse. Total conditioned medium from the multiple cell types, containing a soluble fraction and an exosome fraction, was harvested at each passage and the passages combined into one batch for product development. Parts of our stem cell technology used here are covered by US patents [46–49].

## Safety studies, human repeat insult patch test & canine repeat insult patch test

The canine repeat insult patch test and human repeat insult patch test [24] were used to assess primary and accumulative irritation, and/or allergic contact sensitization, in both male and female human subjects between the ages of 18 and 66. All subjects were free of skin disease and were prohibited from using topical or systemic antihistamines and steroids, beginning 7 days prior to the onset and throughout the duration of the study. Measurements were performed by a trained rater technician. For the induction phase of the study, the patches. 0.1 g of the combinatorial secretome per square inch Webril dressing, were applied three-times weekly for 3 weeks, for a total of nine applications. The patches were removed 48 h after application and evaluated before a fresh patch was reapplied to the same area. For the challenge phase of the study, 2 weeks after the final induction phase patch was applied, an adjacent, virgin area of skin then received a fresh patch and was evaluated at 24 and 72 h following application of the patch. Ratings were scored using the following table:

0 No visible skin reaction.
1 Barely perceptible or spotty erythema.
2 Mild erythema covering most of the test site.
3 Moderate erythema, possible mild edema.
4 Marked erythema, possible edema.
5 Severe erythema, possible edema, vesiculation, bullae and/or ulceration.

For the canine repeat insult patch test study, 27 qualified subjects absent of skin disease or irritation and no use of medications, male and female, ranging from age 2 to 7 years were studied. Stem cell released molecules from the ADSCs and FBs (S2RM) was applied to the belly of the dog, held for 2 min to allow the S2RM to absorb into the skin, and no patch applied. The rater was a practicing veterinarian, who used the same rater scale that was used in the HIRPT. Photos were taken following each application, which was performed three-times weekly for 3 weeks. All 27 dogs finished the study.

## Oral toxicity studies

This toxicity study used three groups of 3 male and 3 female Sprague–Dawley rats (18 total). Once-daily oral (gavage) administration was as follows: Group 1 was administered the vehicle only once daily at 4.4 ml/kg/dose for 28 consecutive days. Group 2 was administered S2RM once daily at 0.44 ml/kg/dose for 28 consecutive days. Group 3 was administered S2RM once daily at 4.4 ml/kg/dose for 28 consecutive days. As a biomarker for a possible inflammatory response to the S2RM administration, all rats were subjected to blood collection (Red-Top) for ELISA testing for the pro-inflammatory cytokines IL-10 and IL-31, which measured prior to the first dose (Day 1) and on the day following the last dose (Day 29).

## Results

### Batch reproducibility: total protein

A shotgun approach to a proteomic analysis has yet to be performed, but in 10 samples from various batches, we measured, using a microarray, 18 proteins in an exosome fraction present in each. The presence of the 18 proteins and exosomes in each batch, along with strict standard operating procedures (SOPs) and low variability in total protein count from batch to batch suggests that each batch is similar. RNA and metabolites have not yet been measured. The Bradford method was used in all protein determinations. The Bradford protein assay is a spectroscopic analytical procedure used to measure the concentration of protein in a solution. Samples, performed in duplicate, from a total of 15 batches of S2RM were analyzed. The variation between duplicates in all cases was less than 10%. Five samples of different S2RM batches and five samples of the individual batches of SRM from the individual cell lines that make-up the S2RM were analyzed for total protein. All samples were taken from frozen aliquots of batches previously used to make skincare products with demonstrated efficacy. All batches produced at least 500 ug/ml of total protein, with a high value in one batch of 630 ug/ml. The mean value of all batches was 554 ug/ml. The variability in all the batches was 20% or less. With the exception of one batch displaying a high protein count (630 ug/ml), the rest of the batches had a variability of 12% or less.

### Skin safety testing, irritation

Totally, 91 participants, both male and female and ranging in age from 18 to 66 years, were selected for this evaluation. A total of 50 participants completed the study. The remaining subjects discontinued their participation for various reasons, none of which were related to the application of the test material. Of the 50 subjects completing the study, all were rated at 0 during both the induction phase and the challenge phase, indicating that the S2RM induced no immediate or long-term irritation, or allergic reaction. Because the S2RM is also intended for therapeutic development to treat companion animals, we tested for cross-species irritation in canines. Similar to that for humans, no irritation or allergic reaction was observed in the 27 canines, as all scored ‘0’ throughout the testing period.

### Analysis of ocular pathology & tumorigeneicity following topical application of S2RM to the eye

Both eyes were recovered from all animals following seven consecutive days of twice daily administration of the S2RM as eye drops applied to the cornea. Eyes were submitted to Colorado Histo-Prep for histopathological evaluation by a board-certified veterinary pathologist who had no knowledge of which eyes had been treated. Six rabbits (New Zealand White) received the S2RM solution in one eye, and the other eye served as a control. One control rabbit receiving no S2RM in either eye was also was also used. The samples were trimmed, processed, embedded, sectioned and stained. Histopathology of the tissues was conducted on slides stained with hematoxylin and eosin with an emphasis on eye irritation and inflammation.

### Oral administration toxicity, tumorigenesis & inflammation results

**Figure 1.**
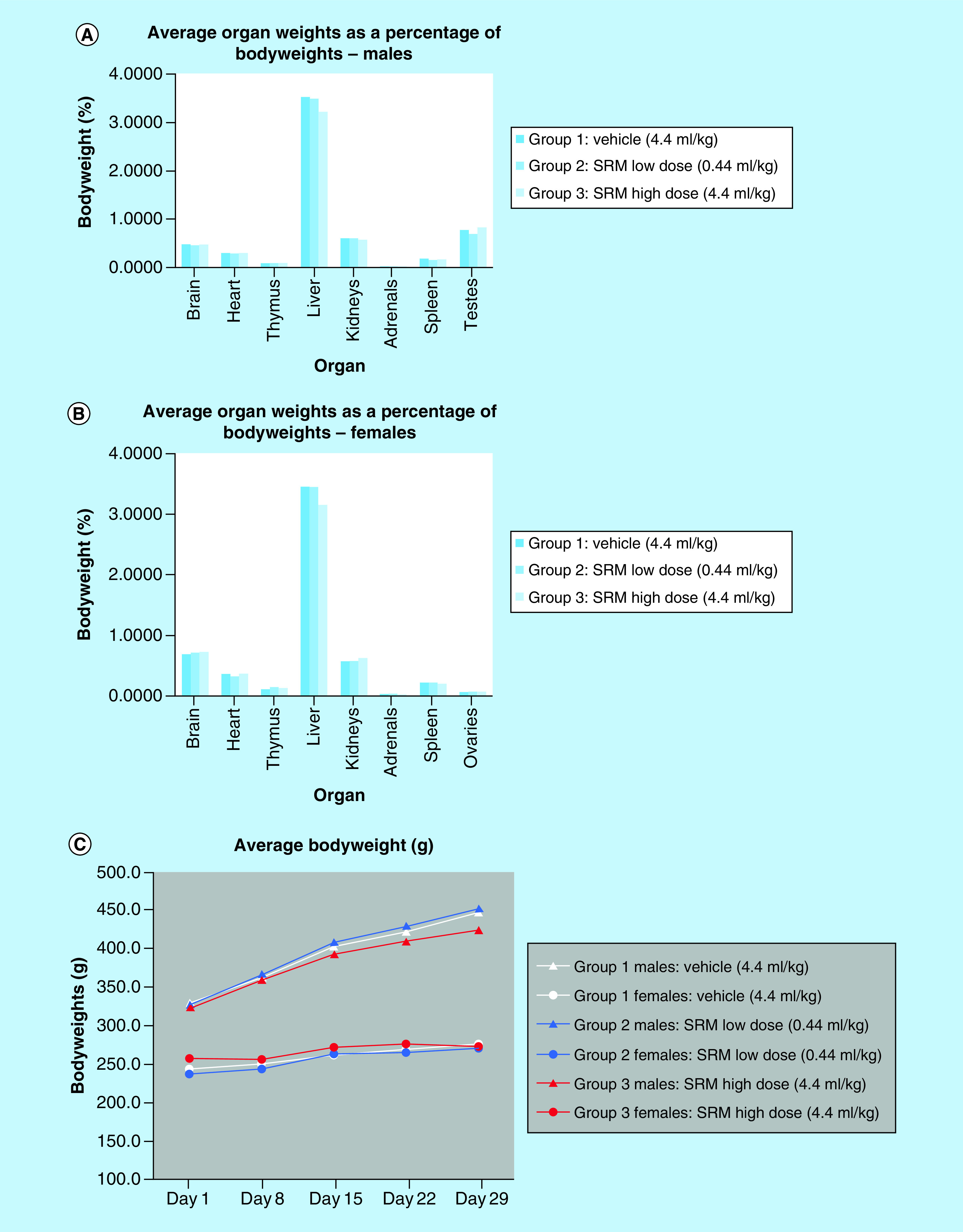
(A) Average organ weights as a percentage of bodyweight for males, and (B) is that for females. **(C)** Total bodyweight over a 30-day period during dosing by oral gavage of S2RM. No significant differences in weights were observed for any of the organs, and none of the organs showed evidence of toxicity or tumorigenesis SRM: Stem cell released molecules; S2RM: Stem cell released molecules from the adipose mesenchymal stem cells and fibroblasts.

No significant differences in organ weights were found in the male or female rats when compared with controls (Figure 1A & B). When compared with the control group, there were no biologically relevant changes from Day 1 to Day 29 for group average IL-10 or IL-31 in either SRM-treated group, suggesting that the S2RM did not induce an inflammatory reaction when administered orally. All tissues were also analyzed for tumor formation, and no tumors were found in any of the analyzed tissues (analyzed tissues are depicted in Figure 1). As a general indicator of health, bodyweights were recorded prior to the first dose and weekly thereafter, including the day of euthanasia (Figure 1C). When compared with the control group, there was mildly decreased bodyweight gain in high-dose (SRM at 4.4 ml/kg/dose) males and females over the course of the study. Bodyweight in the S2RM low-dose group (Group 2, 0.44 ml/kg/dose) was comparable to the control group throughout the study in males and females. Therefore, the general health and the appetite of the experimental animals were normal.

### Results of ocular pathology & tumorigeneicity following topical application of S2RM to the eye

No S2RM-related lesions or tumors were observed in this study after 7 days of topical S2RM/placebo treatment and prior to euthanasia. The eye globe sections were of very good quality and no differences in cornea morphology were detected in any specimen. Overall eye structure was normal, and no ocular opacities were detected.

### Ames test

The Ames reverse mutation was used in four strains of *Salmonella.* Mutagens identified using the Ames test are usually carcinogens, given Ames showed an association of carcinogenicity and mutagenicity of 90%; in other words, most carcinogens are also mutagenic (Table 1) [25]. In the four strains of bacteria tested (Figure 1), the S2RM was shown to be nonmutagenic tested with or without S9 activation. S9 is a crude liver enzyme extract that was used to convert materials without any genotoxic activity to active genotoxic entities; in other words, to duplicate the possible liver derived metabolites of the test product that are mutagenic.

### Cellular cytotoxicity in a mouse L929 FB cell line

**Table 1.** Ames test for mutagenicity in four strains of bacteria shows that the S2RM is not a mutagen.

A. Strain characteristics and standard strain plate counts								
Sector	Characteristics (expected)	TA97a	TA98	TA100	TA1535			
I	Histidine (no growth)	NG	NG	NG	NG			
II	rfa mutation CV (zonal inhibition)	S	S	S	S			
III	+AMP	G	G	G	G			
IV	+AMP and +TET	NG	NG	NG	NG			
N/A	uvrB/uvrA-UV light	NG	NG	NG	NG			
N/A	Titer (organisms/50 μl)	15	59	84	10			
B. Standard plate incorporation assay – reversion rates for tester strains								
	*Salmonella typhimurium*							
	TA97a		TA98		TA100		TA1535	
	CFP	Mean	CFP	Mean	CFP	Mean	CFP	Mean
Negative control without S9	64 51	58	25 26	26	24 52	28	24 21	23
Negative control with S9	65	71	31 34	33	33 26	30	18 18	18
ICR 191 acridine without S9 positive control	163 203	180						
Daunomycin without S9 positive control			156 165	160				
Sodium azide without S9 positive control					67 74	71	44 72	58
Mitomycin C without S9 positive control								
2-aminoanthracene with S9 positive control	358 342	350	430 404	420	155 127	140	93 92	93
Product solution without S9 concentration of 5 mg/plate	85 99 94	93	24 16 18	19	25 31 35	30	17 18 15	17
Product solution with S9 concentration of 5 mg/plate	119 85 103	100	19 40 39	33	26 51 35	37	24 31 21	25

A twofold or greater increase in the number of mean revertants for the S2RM over the mean number of revertants obtained from the negative controls was considered mutagenic.

S2RM: Stem cell released molecules from the adipose mesenchymal stem cells and fibroblasts.

Blank cells indicate not applicable.

CFP: Counts from plate; G: Growth; Mean: Average of plate; NG: No growth; S: Sensitive; S2RM: Stem cell released molecules from the adipose mesenchymal stem cells and fibroblasts.

FBs exist in most tissue compartments throughout the body, particularly in the skin where the current S2RM technology has proven to be efficacious in a number of skin conditions [7]. We, therefore, tested the S2RM for potential cytotoxic effects in FBs. Table 2 demonstrated the data providing evidence that the S2RM has no cytotoxic effects in FBs as measured by observing changes in cell structure or the number of cells in the culture.

**Table 2.** Using a mouse cell line L929, the S2RM conditioned media is shown to have no direct cytotoxic effects on the cells (fibroblasts).

Sample	Malformation	Degeneration	Sloughing	Lysis	Reduction in cell layer density
Sample – 24 h	0.0	0.0	0.0	0.0	0.0
48 h	0.0	0.0	0.0	0.0	0.0
Negative control – 24 h	0.0	0.0	0.0	0.0	0.0
48 h	0.0	0.0	0.0	0.0	0.0
Media control – 24 h	0.0	0.0	0.0	0.0	0.0
48 h	0.0	0.0	0.0	0.0	0.0
Positive control – 24 h	2.2	2.2	2.2	2.2	2.4
48 h	4.4	4.4	4.4	4.4	4.4

Evaluation of results: after incubation, the biological reactivity (cellular degeneration and malformation) is described and rated on a scale of 0–4 as follows: grade reactivity description of reactivity zone. 0, none (no detectable zone under or around specimen); 1, slight (some malformed or degenerated cells under specimen); 2, mild (zone limited to area under specimen); 3, moderate (zone extends 0.5 to 1.0 cm beyond specimen); 4, severe (zone extends greater than 1.0 cm beyond specimen but does not involve the entire dish). If both test articles exhibit reactivity grades of 0, 1 or 2, the sample is noncytotoxic. If both test articles exhibit reactivity grades of 3 or 4, the sample is cytotoxic. If only one (1) test articles exhibits a grade 3 or 4, the test may be repeated with four (4) test articles. To be noncytotoxic, none of the four retest articles may exhibit a grade of 3 or 4.

S2RM: Stem cell released molecules from the adipose mesenchymal stem cells and fibroblasts.

### Protein misfolding tests (Dermal Irritection^®^ Assays System)

In this study, the Irritection^®^ system from InVitro International (CA, USA) was used for the detection of misfolding and structural changes in proteins. Using this methodology, an irritant chemical will disrupt the ordered structure of keratin and collagen and result in the release of a bound indicator dye. Additionally, irritants will induce changes in the conformation of the globular proteins found in the reagent solution. The extent of dye release and protein denaturation was quantitated by measuring the changes in the optical density of the S2RM solution at 450 nm (OD450). The results provide evidence that S2RM induced no structural changes or misfolding of the test proteins (Table 3).

### Heavy metals

Numerous products and procedures are involved in the processing of the stem cells for the collection of their molecules, presenting a number of points were contamination may take place. We, therefore, tested for the presence of four common heavy metals in the S2RM end-product used as the therapeutic. No significant level of heavy metals was present in the S2RM (Table 4).

## Discussion

The results presented here for a combination therapeutic containing the stem cell-released molecules from ADSCs and FBs provide evidence that these molecules are safe whether they are administered orally or topically to the eye or skin. No signs of toxicity, tumorigenesis, mutagenicity, irritation and allergic reaction or inflammation were measured when the S2RM was compared with controls. These data are also important for stem cell transplants because when transplanted stem cells graft in the tissue and remain alive and healthy for a long time [26], much of the cell’s therapeutic effect is mediated by the release of molecules that act as paracrine and autocrine factors [27,28]. Understanding the cellular effects versus the paracrine effects of stem cell therapy is important for the development of therapeutics, both in terms of efficacy as well as safety. For example, we know that BMSC transplants induce aging of the implanted tissue [14], and increase the probability of cancer [8,29], but we do not understand the mechanisms by which this happens. Is it the choice of the BMSC type versus the ADSC type that is responsible for these issues [30], or the implantation process of exogenous cells, or the release of specific molecules from the BMSCs that cause these adverse events? Although we have no evidence to answer the aforementioned questions directly, in this study, we do provide an evidence that the molecules from ADSCs and FBs are safe for therapeutic development. To help answer these questions, other studies have shown that the secretome from BMSCs induces tumorigenesis in an *in vivo* and *in vitro* mouse model through activation of mTOR [31], whereas ADSCs and their conditioned media inhibit cancer growth using *in vivo* and *in vitro* mouse models [32], suggesting that ADSCs are a better cell choice for therapeutic development than are BMSCs in terms of tumorigenesis. ADSCs also present numerous other benefits compared with BMSCs for a variety of safety and efficacy issues [15]. However, more data are needed to understand the long-term consequences of the S2RM on various health parameters, and whether injection and intravenous (IV) administration will present a like safety profile as observed in the current studies.

In the development of stem cell therapeutics, Goldring *et al.* [33] asked the question of whether we are setting a higher bar for the clinical implementation of stem cell-derived therapeutics than we currently apply for other types of cellular therapy. The authors argue there is a danger that if perfection is a prerequisite for beginning stem cell therapeutics, then we will never begin. As Maguire [30] has argued, if the current hype about stem cell therapies continues, where unapproved therapies without a knowledge of risk or reward are burgeoning [34], without a knowledge of the procedure’s risks, then a proper evaluation of the risk versus reward ratio cannot be made for that particular stem cell therapy. Regardless of where the bar is set for other therapies, where medical procedures, like most drugs, have unknown or hidden long-term consequences to health, the risks must be evaluated as best as possible so informed risk versus reward decisions can be made. Often, when considering marketed drugs, not until Phase IV postmarket approval, are the long-term consequences of a drug discovered. This is exemplified by the many drugs pulled from market or with safety issues 3–4 years after their approval [35,36]. Even more unfortunate, the problem is worse with medical procedures [37]. Such is the case with approved stem cell transplants. Many case studies have reported the approved stem cell transplants to be associated with the later development of cancer [8], and unapproved stem cell transplant procedures are notorious for adverse side-effects, including development of cancer [38]. The effects of approved BMSC transplant in cancer relapse are not well understood but are thought to involve epigenetic factors in the stem cells used for the transplant [29]. In addition, BMSC transplants may cause aging of the tissue as measured in T cells using a p16 biomarker [14], indicating the increased level of cellular senescence in the surrounding tissue, a factor in the increased probability of tumorigenesis [39].

Although stem cell therapy is in a period of rapid advancement, the science of stem cell safety assessment must also evolve, not to hinder progress, rather to support, guide and expedite the progress of patient treatment using stem cell-based technologies. The development of a rich safety database is necessary to ensure that we can proceed with appropriate safeguards in place and allow that stem cell-based therapeutic approaches develop in a way that benefits society overall by using well supported, data-driven risk versus reward analysis. These data presented here are one such needed set of safety data to evaluate the patient risk versus benefit ratio for stem cell therapeutics in general, and specifically for our combination of stem cell released molecules from ADSCs and FBs.

**Table 3.** S2RM did not induce any misfolding or structural changes in the collagen, keratin and other dermal proteins.

Sample	Dose	IDE Score	Predicted ocularIrritancy classification
S2RM	25 μl	9.6^A^	Minimal irritant
S2RM	50 μl	7.1	Minimal irritant
S2RM	75 μl	5.5	Minimal irritant
S2RM	100 μl	4.8	Minimal irritant
S2RM	125 μl	3.0	Minimal irritant
A. Maximum qQualified score			
IDE	Predicted ocular irritancy classification		
0.0–12.5	Minimal irritant		
12.5–30.0	Mild irritant		
30.0–51.0	Moderate irritant		
51.0–80.0	Severe irritant		

Irritant level is the degree of misfolding and structural change in the protein content of the S2RM.

IDE: Irritection draize equivalent; S2RM: Stem cell released molecules from the adipose mesenchymal stem cells and fibroblasts.

**Table 4.** Testing for the presence of four common heavy metals using inductively coupled plasma mass spectrometry.

Test	Result
Arsenic	<0.025 p.p.m.
Cadmium	<0.025 p.p.m.
Lead	<0.025 p.p.m.
Mercury	<0.025 p.p.m.

None of the heavy metals tested were present in significant, unsafe amounts.

p.p.m.: Part per million.

## Future perspective

To date, stem cell therapeutics have heavily focused on using stem cell transplants. However, for many conditions where stem cells have been used as the therapeutic, most of the therapeutic benefit of the stem cell transplant was because of the many molecules released by the transplanted stem cells. Many medical procedures have little or no reward, and only present risk to the patient [37]. Indeed, many stem cell transplants procedures have inherent negative consequences to the patient, including a number of safety issues.

The molecules and exosomes released from certain stem cell types will provide a more efficacious, safe, less expensive and easier to dose therapeutic than do stem cells for a number of conditions. In the next decade, we are likely to optimize the methods to store and process the exosomes from stem cells, such as freezing methods [40] and lyophilization [41,42]. This knowledge will help to facilitate the formulation, transportation, and ‘off the shelf’ use of exosomes for therapeutic application, obviating many of the difficulties associated with cellular therapies [43]. Given that molecules released from specific stem cell types, such as BMSCs and ADSCs, and their various phenotypes, can preferentially elicit inflammatory, infection fighting pathways (BMSCs) or proresolving, wound healing pathways (ADSCs) [15], the choice of stem cell type and its specific phenotype will be critical to their therapeutic development for particular diseases and conditions. For example, in many viral diseases, including COVID-19, the disease is initially characterized by high viral loads in tissues, such as pulmonary and cardiovascular tissues, followed by clearance of the virus. This can lead to sequelae after the viral clearance, due a dysregulated innate and adaptive immune response, which can include a life threatening ‘cytokine storm.’ Given that adult stem cells are part of and regulate the innate and adaptive immune systems [44], and that some stem cell types are better at fighting infection, while other types are better at resolving inflammation and repairing tissue, a therapeutic regimen for COVID-19 may include an early phase dosing of exosomes from BMSCs to fight infection [15], and a later phase dosing of exosomes from ADSCs once the virus has cleared to resolve inflammation and rebuild the damaged tissue, with the potential to reduce fibrosis [15] in the alveoli and heart tissue [45].

Given there are currently no approved exosome therapies, future work will need to test these ideas in animal models that best predict outcomes in humans, and to strategize with the Federal Drug Administration, EMA and other regulatory bodies to develop the best chemistry, manufacturing and controls methods and clinical tests to bring these stem cell released molecules/exosomes to the market as approved therapeutics for defined health indications.

**Summary points**

- A combination of the molecules from adipose mesenchymal stem cells and fibroblasts were tested for their safety as a therapeutic.
- A Repeat Insult Patch Test in humans demonstrated no irritation.
- No ocular pathology or tumorigeneicity was observed.
- Oral administration resulted in no tumorgeneisis, toxicity, or inflammation.
- The Ames test for mutagenicity was negative.
- There was no cellular toxicity in a fibroblast cell line.
- No misfolding or structural changes in collagen and keratin proteins was measured.
- Mass spectrometry analysis measured no significant amount of heavy metals.
